# Relationship of Body Mass Index to Alcohol Consumption in College Freshmen

**DOI:** 10.1100/2012/849018

**Published:** 2012-05-02

**Authors:** Mary A. Nies, Linman Sun, Donna Kazemi, Amy Carriker, Jacek Dmochowski

**Affiliations:** College of Health and Human Services, University of North Carolina at Charlotte, 9201 University City Boulevard, CHHS 416, Charlotte, NC 28223, USA

## Abstract

*Objective*. Assess the relationship between body mass index (BMI) and drinking in college freshman. *Method*. College freshman (*N* = 199) at a university completed the drinking questionnaires. Drinking amount and the alcohol problem index (RAPI) served as outcomes, and BMI was the independent variable. *Results*. RAPI scores were associated with gender, amount of drinking, and BMI (*P* < 0.001, *F* = 13.44). Increase of RAPI with drinking amount was larger for females (slope = 0.06) than for males (slope = 0.03). *Conclusion*. This information can be helpful when providing health promotion strategies to college students regarding nutrition modifications that would be most beneficial for their health.

## 1. Introduction

This study investigated the relationship among body mass index (BMI), stages of change, number of drinks, and consequences of drinking in college freshmen. Consumption of alcohol is high among college students. According to Lloyd-Richardson et al. [1], 63% of students reported consuming alcohol within the past 30 days, and 42% report either excessive drinking or binge drinking. These researchers suggested that of the students who did drink alcohol, 67% did not know the number of calories they were consuming in the alcohol.

 The amount and frequency of alcohol consumed have been found to correlate with BMI. In a large cross-sectional survey, noninstitutionalized individuals were administered a questionnaire to determine their history of drinking, quantity of drinking, frequency of drinking, average volume of drinking, and binge drinking [2]. The binge drinkers and the heavy drinkers often had a significantly higher BMI than those who participated in only light-to-moderate drinking. The National Institute on Alcohol Abuse and Alcoholism's [3] National Advisory Council defined binge drinking as five or more drinks in about 2 hours for a male and four or more drinks for a female. A study by Breslow and Smothers [4] using national surveys in the United States examined quantity, frequency, and average volume to categorize alcohol consumption based upon participant information. They found that people who consumed four or more drinks per day had significantly higher BMIs than those who consumed one drink per day.

Frequency of drinking has sometimes been found to have an inverse relationship with BMI. Studies have suggested that people who drink a small amount daily have a lower BMI. Arif and Rohrer [2] and Breslow and Smothers [4] reported that the participants in their study who frequently consumed a small amount of alcohol had lower BMIs than participants who drank heavily or binged regularly. Breslow and Smothers [4] also found that the participants who drank the most frequently had lower BMIs than those who drank less frequently. The benefits of drinking, however, decreased after two drinks per day, when BMI started to increase. Vadstrup et al. [5] reported a relationship between the type of alcohol consumed and waist circumference after 10 years. Participants who drank beer had a higher waist circumference, whereas those who consumed wine tended to have a lower waist circumference.

 Data on the relationship of BMI to alcohol consumption in the college-age population have been limited. The role of BMI as an independent variable on college student drinking as an outcome variable remains unclear. Additional studies are needed to explore the relationship of BMI to the number of drinks and gender in this population. This study attempted to help fill this gap in the literature by assessing the relationship among BMI, stages of change, number of drinks, and consequences of drinking in college freshmen. Drinking amount and related negative consequences served as outcomes; BMI was the independent variable.

## 2. Methods

### 2.1. Setting and Design

This descriptive, correlational study used a convenience sample of 199 college freshman males and females ages 18 to 20 at a large southern university who indicated that they had more than zero drinks for a typical week. Institutional Review Board was approved, and following completion of the informed consent, the participants completed the questionnaires in a private room.

### 2.2. Measures

 The following measures were completed: daily drinking questionnaire (DDQ) [6]; the brief readiness to change questionnaire (BRCQ) [7]; the Rutgers alcohol problem index (RAPI) [8]. Self-reported BMI was calculated from height and weight, and demographic data were collected. Drinking amount and the RAPI served as outcomes; the BMI was the independent variable.

#### 2.2.1. BMI

BMI is a measurement that combines a person's weight and height. BMI is used to estimate a healthy body weight based on the height. It is widely used to diagnose whether individuals are underweight, overweight, or obese. BMI is defined as body weight divided by the square of one's weight. The formula used is a unit of measure of kg/m^2^. A BMI of 18.5 to 25 may indicate optimal weight, a BMI lower than 18.5 suggests underweight, and a BMI above 25 may indicate overweight. A BMI below 17.5 may indicate anorexia nervosa or a related disorder, and a BMI above 30 suggests obesity, with a BMI above 40 indicative of morbid obesity [9].

#### 2.2.2. DDQ

All of the participants completed the DDQ. They reported the number of drinks consumed on that day of a typical week during the past month. The number of drinks was calculated by summation, that is, by the total number of drinks consumed weekly. A drink is equal to a 12oz beer or a 1oz shot, or a 4oz glass of wine.

#### 2.2.3. BRCQ

The BRCQ is a 12-item questionnaire. It is a brief measure of motivation to change alcohol use developed to be consistent with the transtheoretical stages of change model [10]. The BRCQ, which requires 2 minutes to complete, assigns drinkers to one of three stages: precontemplation (P), contemplation (C), or action (A). The BRCQ yields stage-scale scores for P, C, and A, as well a total readiness to change score (−24 to 24) derived by the formula (C + A)-P. A negative-scale score reflects disagreement with items measuring the stage of change; a positive score represents agreement. The highest scale score represents the stage of change designation. For the current study, when the two scale scores were equal, the researchers chose the scale farther along the continuum of change. High scores on the P scale signified a lack of readiness to change, whereas high scores on the A scale signified a readiness to change [7].

#### 2.2.4. RAPI

The RAPI is a brief 23-item measure with good psychometric properties designed to assess the frequency of the occurrence of negative drinking consequences [8]. The RAPI, which has been used successfully with college student samples, seeks information about the experience of consequences over the last year [11]. The RAPI requires approximately 5 minutes to complete. Participants circle the number of times they have experienced each problem on a 4-point scale within the last year (0: *none*, 1: *1-2 times*, 2: *3*–*5 times*, and 3: *more than 5 times*). The final RAPI score is the sum of the responses. The range for the RAPI score is 0 to 69. High scores indicate several alcohol problems. In a clinical sample of adolescents ages 14 to 18, the means ranged from 21 to 25; in a nonclinical sample ages 15 to 18, means ranged from 4 to 8, depending upon age and sex.

### 2.3. Data Analysis

Data analysis was completed using SAS 9.2. Thirty nine percent of the participants were females; 54.2% of the participants were in the P stage, 14.3% in the C stage, and 31.5% in the A stage. Average BMI for females and males was 24 kg/m^2^. Participants consumed an average of approximately 13.5 drinks per week; the mean RAPI score was 11 (see Table 1).

Multiple regression was used to assess the association among number of drinks (per week), the RAPI score, and BMI, with adjustment for gender and readiness to change stage. Residual diagnostics were performed for the final models used in the analysis.

## 3. Results

**Figure 1:**
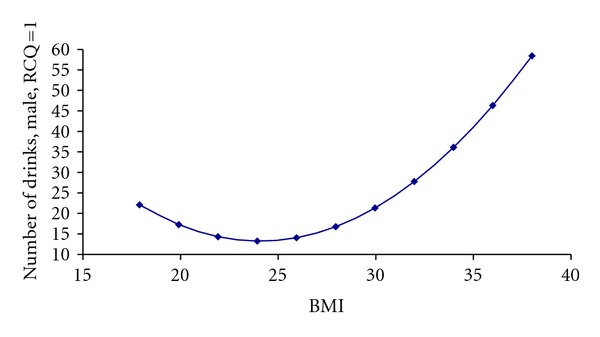
Association between number of drinks and BMI for males in precontemplation stage.

Drinking was associated with gender, BMI, and stage of change (*P* < 0.0001 for the whole model). For males precontemplating change, the relationship between BMI and amount of drinking was nonlinear. Participants with average BMIs drank the least (i.e., about 12 drinks per week), and those with the highest BMIs drank the most (i.e., almost 50 drinks). Participants with the lowest BMIs fell in between the range (i.e., about 21 drinks) (see Figure 1 and Tables 2 and 3).

**Figure 2:**
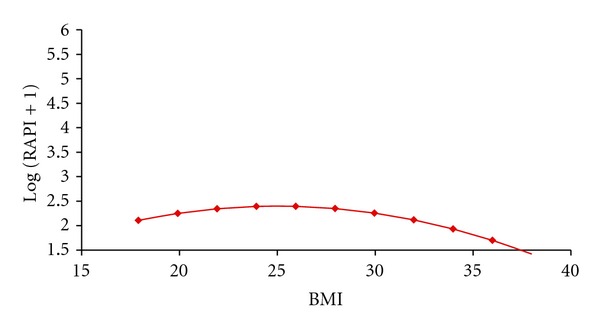
Association of RAPI to BMI.

**Figure 3:**
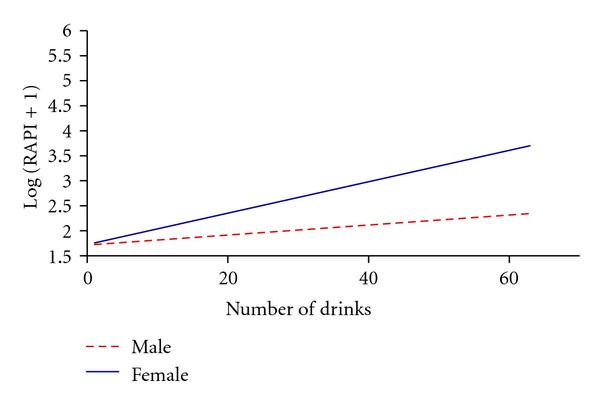
Association of RAPI to drinking amount and gender.

RAPI scores were associated with gender, amount of drinking, and BMI (*P* < .001, for the whole model). Increase of RAPI with drinking amount was larger for females (slope = 0.06) than for males (slope = 0.03). Participants with high BMIs had lower RAPI scores (see Figures 2 and 3 and Table 3).

## 4. Conclusion

 The relationship between BMI and number of drinks was significant only for the male participants in the P stage, that is, the male participants in the P stage with high BMIs drank more. These male participants were not taking any action to change. The male and female participants who were obese had fewer alcohol consequences; however, drinking was still harmful to their health. Given the number of drinks consumed, the male participants experienced fewer consequences than the female participants did.

## 5. Discussion

 The male participants in the P stage were not thinking about making any changes, so those with the highest BMIs may have felt comfortable consuming more alcohol. Even though they had high BMIs, the male participants in the C and A stages did not consume more alcohol because they were trying to make changes to their drinking behaviors. For the female participants, the stages of change did not have a significant effect.

 The more alcohol that the students, male and female, drank, the more alcohol-related consequences they encountered; however, the male participants had a greater ability to handle the consequences perhaps because males have more muscle tissue and can absorb alcohol more efficiently than females can. Thus, it is important to focus on developing interventions for females.

 This information can be helpful to clinicians who are striving to provide health promotion strategies to college students regarding nutrition modifications that would be the most beneficial to their health. Alcohol is high in calories, so drinking alcohol may contribute to higher BMIs and the poor health choices of college-age students who are already overweight, particularly freshmen female students who drink four or more drinks per day and male students who consume five or more drinks per day. College students should be cautioned about the high carbohydrate content of alcohol, especially if they drink large amounts. Elevated triglycerides and low high-density lipoprotein cholesterol levels are the result of continued high carbohydrate intake, especially if students are already overweight when they first start to drink [12]. Moderation in drinking alcohol should be discussed with student in an effort to prevent an increase in BMIs and problems associated with drinking.

## Figures and Tables

**Table 1:** Basic characteristics of participants (*N* = 199).

Whole sample	*N*	No. of drinks range		No. of drinks *M*	No. of drinks SD	BMI range		BMI *M*	BMI SD	RAPI range		RAPI *M*	RAPI SD
	199	1	63	13.46	10.57	17.93	37.99	23.98	3.63	0	47	10.95	8.47
RCQ = 1	107	1	49	12.25	9.48	17.93	37.99	24.23	3.80	0	38	8.20	6.79
RCQ = 2	28	3	44	18.90	10.24	18.55	30.65	23.77	3.55	5	32	18.07	8.12
RCQ = 3	64	1	63	13.08	11.83	18.00	36.24	23.65	3.37	0	47	12.45	9.06
Male	78	1	63	18.75	12.78	17.93	36.24	24.37	3.56	0	47	10.58	8.15
RCQ = 1	44	1	49	15.85	10.85	17.93	34.57	24.38	3.50	0	20	7.98	4.96
RCQ = 2	12	9	44	25.73	10.71	19.89	28.33	23.06	2.56	10	32	18.54	8.18
RCQ = 3	22	1	63	20.43	15.78	18.12	36.24	25.05	4.06	0	47	11.09	10.22
Female	121	1	34	10.09	7.11	18.00	37.99	23.73	3.66	0	38	11.19	8.69
RCQ = 1	63	1	34	9.85	7.63	19.00	37.99	24.14	4.02	0	38	8.35	7.81
RCQ = 2	16	3	21	13.34	5.52	18.55	30.65	24.31	4.15	5	32	17.69	8.32
RCQ=3	42	1	26	9.23	6.57	18.00	29.94	22.91	2.72	0	37	13.17	8.43

**Table 2:** Association of number of drinks With BMI for males in precontemplation stage^1^.

Parameter	Estimate	SD	*t* value	95% CI		Pr>|*t*|
Intercept	148.01	54.8	2.7	39.88	256.14	0.008
BMI	−11.21	4.33	−2.59	−19.75	−2.66	0.01
BMI ∗ BMI	0.23	0.08	2.75	0.07	0.4	0.007

^1^All the other terms in the model allowing quadratic dependence on BMI for combinations of gender with RCQ status were not significantly dependent on BMI.

**Table 3:** Association of RAPI with BMI adjusted for number of drinks and gender.

Effect	Estimate	SD	*df*	*t*	*P*	95% CI
Intercept	−1.91	1.78	193	−1.08	0.28	(−5.42,1.59)
male	0.01	0.19	193	0.08	0.94	(−0.36,0.39)
Drinks	0.06	0.01	193	6.2	<.0001	(0.04,0.08)
Drinks ∗ male	−0.03	0.01	193	−2.57	0.01	(−0.05, −0.01)
BMI	0.29	0.14	193	2.08	0.04	(0.02,0.56)
BMI ∗ BMI	−0.01	0.00	193	−2.19	0.03	(−0.01,0.00)
